# 344. VersaTrek versus Virtuo Blood Culture System’s Effect on Diagnostic Test Utilization and Patient Outcomes, a Quasi-Experimental Study

**DOI:** 10.1093/ofid/ofac492.422

**Published:** 2022-12-15

**Authors:** William P Dillon, Naoshin Khan, Dana Nachawati, George J Alangaden, Linoj Samuel, Mayur Ramesh

**Affiliations:** Henry Ford Hospital, Detroit, Michigan; Henry Ford Hospital, Detroit, Michigan; Henry Ford Hospital, Detroit, Michigan; Henry Ford Health, Detroit, Michigan; Henry Ford Hospital, Detroit, Michigan; Henry Ford Hospital, Detroit, Michigan

## Abstract

**Background:**

Prior studies evaluating the sensitivity blood cultures utilizing the Virtuo blood culture system versus the VersaTREK blood culture system have suggested increased sensitivity with the Virtuo system. Studies evaluating if this increased sensitivity effects patient outcomes are lacking. This study evaluates the effect of replacing the VersaTREK system with the Virtuo system on morbidity and mortality in patients with *Staphylococcus aureus* bacteremia.

**Methods:**

This quasi-experimental study was performed at Henry Ford Health System in Southeast Michigan. We analyzed all patients with positive blood cultures for *Staphylococcus aureus* between 3/1/18 and 9/30/18 (VersaTREK system) and 3/1/19 and 9/30/19 (Virtuo system). The VersaTREK arm had 267 patients and the Virtuo arm 404. Patients transferred to or from outside facilities with ongoing bacteremia were excluded. Primary outcomes were differences in length of stay, mortality at 14, 30, and 90 days from completion of therapy, and hospitalization for recurrence of bacteremia within 30 days. Secondary outcomes were differences in length of therapy, rates of diagnostic imaging tests to assess for persistent foci of infection or an unknown source of persistent bacteremia, and length of bacteremia. Data were analyzed using SPSS for Macintosh using Pearson’s chi-square test and Student’s t-test. A *p* < 0.05 was considered statistically significant.

**Results:**

Demographics of the study populations were equal however, the Charlson Comorbidity Index for VersaTREK arm compared to Virtuo (3.5 to 3.1) trended toward significance (*p*=0.05) (Table 1). There was no difference in length of bacteremia, lengths of antibiotic therapy, CT scans, or echocardiograms performed. More MRIs were performed in the Virtuo arm (*p*=< 0.001) (Table 2). There was no difference in recurrence of bacteremia, patient death due to bacteremia, length of hospital stay, or 30 day readmission for recurrence of bacteremia (Table 3).

**Figure:**
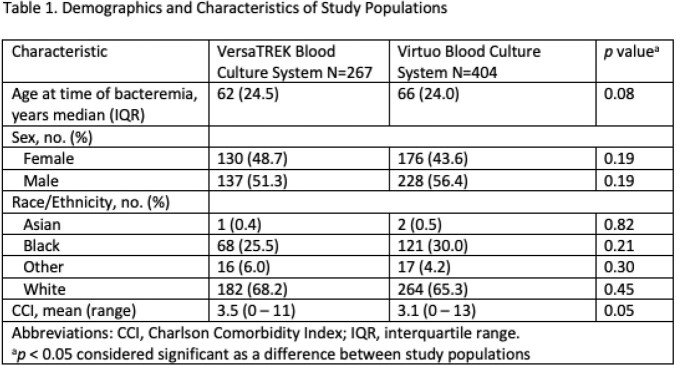

**Figure:**
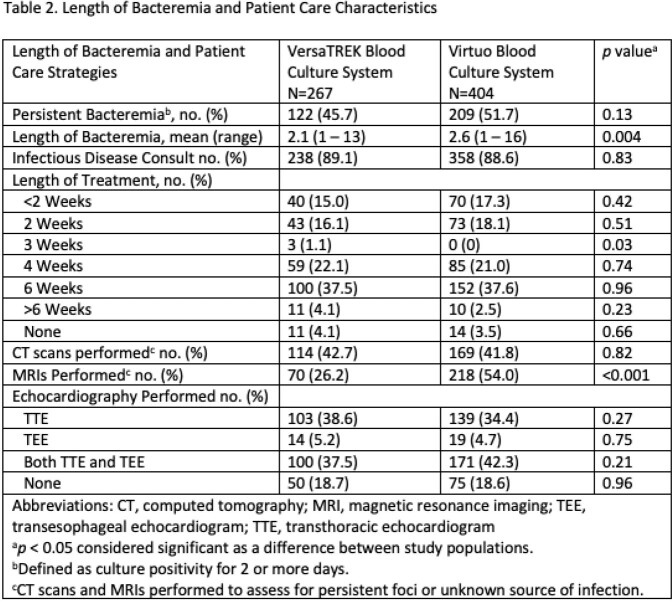

**Figure:**
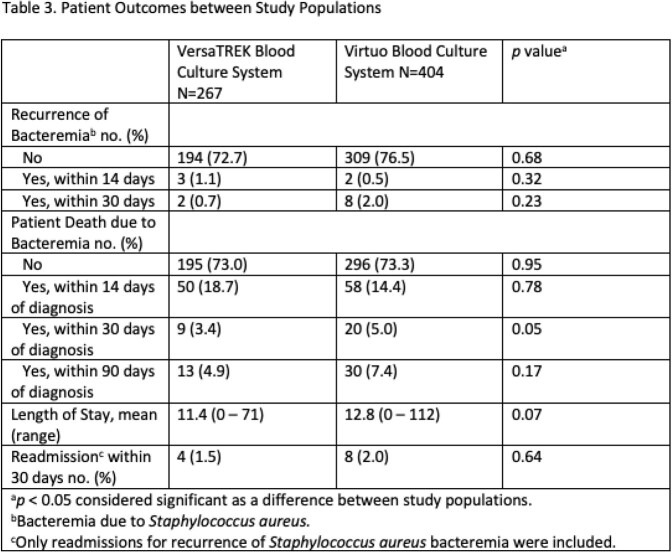

**Conclusion:**

This is the first study to evaluate how increased sensitivity of a blood culture system effects patient outcomes. Our results suggest that *Staphylococcus aureus* bacteremia may be over treated in blood cultures systems that are highly sensitive. Further prospective studies are needed to explore this further.

**Disclosures:**

**All Authors**: No reported disclosures.

